# Disseminated cryptococcal disease during treatment with idelalisib and corticosteroids for follicular lymphoma

**DOI:** 10.1136/bcr-2020-235216

**Published:** 2020-07-05

**Authors:** Paul J Hengeveld, Eva de Jongh, Peter E Westerweel, Mark-David Levin

**Affiliations:** 1 Immunology, Erasmus MC, Rotterdam, The Netherlands; 2 Internal Medicine, Albert Schweitzer Hospital, Dordrecht, The Netherlands

**Keywords:** cryptococcosis, cryptococcus, haematology (drugs and medicines), malignant disease and immunosuppression, haematology (incl blood transfusion)

## Abstract

A patient on a regimen of idelalisib and corticosteroids for a relapse of follicular lymphoma presented to our emergency ward with a fever of unknown origin. Despite the initiation of broad-spectrum antibiotics and fluids, the patient’s clinical condition deteriorated. Eventually, a diagnosis of disseminated cryptococcosis was established and immunophenotyping revealed complete absence of circulating B and CD4^+^-T lymphocytes, and a markedly diminished CD8^+^-T lymphocyte count. In this case, treatment with idelalisib and corticosteroids likely resulted in profound lymphopenia and the first reported instance of disseminated cryptococcosis under this regimen. After the withdrawal of idelalisib and steroids and initiation of antifungal therapy, lymphocyte counts partially recovered. After clinical improvement, the patient could be discharged from the hospital. This case highlights that the combination of idelalisib and corticosteroids can cause significant immunocompromise and opportunistic infections. Additionally, we illustrate the rate of lymphocyte reconstitution after withdrawal from idelalisib and corticosteroids.

## Background

The introduction of small molecular inhibitors that specifically target intracellular signalling pathways on which cancers depend has led to increased life expectancy for patients with indolent B cell malignancies, such as follicular lymphoma (FL).^1^ However, targeted therapies can significantly compromise the immune system, especially in patients who have previously received chemoimmunotherapy and corticosteroids. Consequently, patients with a history of treatment for indolent B cell malignancy are at increased risk for opportunistic infections. In this case report, we describe a patient on idelalisib and prednisone treatment for FL, who developed disseminated cryptococcal disease, an infection with significant mortality usually encountered in patients with compromised T cell immunity.^2^ To the best of our knowledge, this is the first reported case of cryptococcal infection during treatment with idelalisib.

## Case presentation

A 59-year-old man, with a medical history of paroxysmal atrial fibrillation, was diagnosed in 2008 with grade I follicular lymphoma, Ann Arbor stage III, FLIPI 3, with progressive cervical, mediastinal and intra-abdominal lymphadenopathy. After eight cycles of R-CVP (rituximab, 375 mg/m^2^ intravenously, day 1; cyclophosphamide, 750 mg/m^2^ intravenously, day 1; vincristine 1.4 mg/m^2^ intravenously, day 1 and prednisone 40 mg/m^2^ orally, day 1–5), the patient achieved a partial remission. His disease relapsed in 2013 with progressive para-aortal lymphadenopathy, for which he was treated with rituximab (375 mg/m^2^ intravenously, day 1), bendamustine (90 mg/m^2^ intravenously, day 1–2) and lenalidomide (20 mg orally, day 3–21) in the HOVON 110 FL trial. Due to persisting rituximab infusion-related reactions, treatment was abrogated after four cycles and rituximab maintenance therapy was withheld. At this point, the FL was in complete remission.

In 2017, a second relapse occurred with lymphadenopathy, splenomegaly and diffuse bone marrow infiltration. R-CVP was reinitiated, but again severe infusion-related toxicity after rituximab administration occurred. After three courses of CVP without rituximab, he developed severe autoimmune hemolytic anaemia (AIHA), characterised by a haemoglobin concentration of 3.5 g/dL (normal reference range=13.8–17.2) and a markedly elevated reticulocyte percentage (24%, normal reference range=0–2.5), total bilirubin (98 µmol/L, normal reference range <17) and lactate dehydrogenase (319 E/L, normal reference range <250). A direct antiglobulin test was strongly positive for IgG-mediated haemolysis. The AIHA was interpreted as a paraneoplastic phenomenon, pointing to chemotherapy-refractory disease.

After immediate management with prednisone (1 mg/kg orally once daily) and intravenous immunoglobulins (IVIGs; 300 mg/m^2^), treatment with idelalisib (150 mg orally two times per day) was initiated in conjunction with prophylactic trimethoprim-sulfamethoxazole (480 mg orally one time per day) and valaciclovir (500 mg orally two times per day). The AIHA was successfully controlled with this therapeutic regimen and prednisone could gradually be discontinued. However, after briefly withholding idelalisib due to a drug-induced skin rash, haemolysis swiftly reoccurred. Idelalisib was restarted in a dose of 150 mg two times per day, with concomitant high dosed prednisone (1 mg/kg, 90 mg one time per day, which could gradually be tapered to 40 mg daily) to suppress skin toxicity. With this regimen, the patient’s lymphoma, AIHA and skin toxicity remained well-controlled with a very acceptable quality of life.

The current episode began in July 2019, when the patient was admitted to our hospital with a 1-day history of fatigue and fever up to 40°C. Physical examination and vital signs were unremarkable.

## Investigations

Chest radiography, ECG and urinalysis revealed no significant abnormalities. At this time, blood counts showed a stable haemoglobin level at 10.0 g/dL and a leucocyte count of 5.4×10^9^ cells/L (normal reference range=4–11). Leucocyte differentiation showed a normal neutrophil count, but significant lymphopenia (0.3×10^9^ cells/L, normal reference range=1.0–4.8). C-reactive protein (CRP) level was significantly increased at 173 mg/L (normal reference range <10). Blood cultures obtained at initial presentation did not reveal the growth of any organisms. The patient was admitted to our hospital due to suspected sepsis, and intravenous fluids and ceftriaxone were administered. Urinary cultures produced an amoxicillin-sensitive *Enterococcus faecalis,* and the patient was discharged with oral antibiotics for an assumed urinary tract infection.

**Figure 1:**
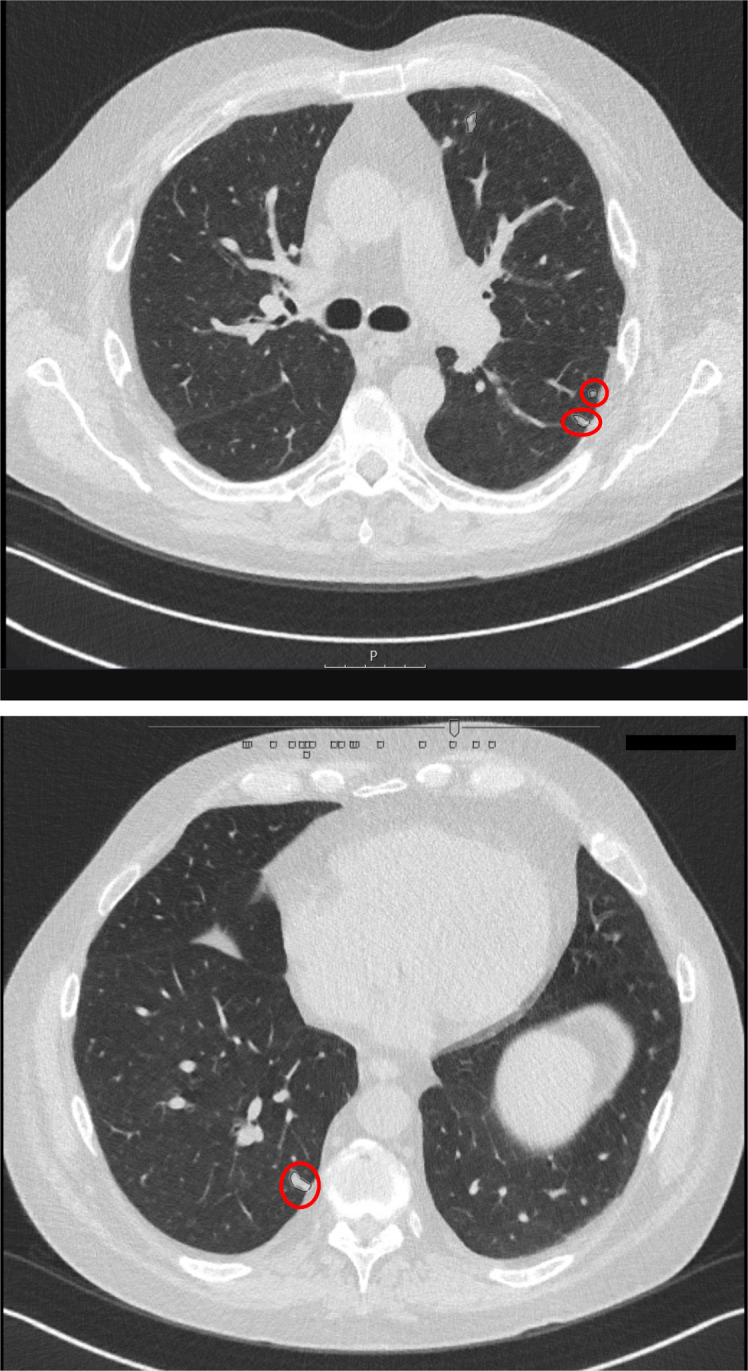
HRCT pulmonary imaging. Two representative stills from the HRCT pulmonary imaging obtained in this patient. The red circles mark intrapulmonary nodular opacities, suggestive of pulmonary cryptococcal disease. HRCT, high-resolution CT.

Two days later, however, the patient was readmitted because of persistent fatigue, malaise and fever, refractory to the prescribed antibiotics. At this time, leucocyte count had fallen to 2.6×10^9^/L and would continue to drop to a nadir of 1.4×10^9^/L over the coming days. Antimicrobial therapy was empirically switched to piperacillin/tazobactam. Blood cultures revealed the growth of *Cryptococcus neoformans*. Although the patient did not exhibit any symptoms suggestive of the central nervous system (CNS) or pulmonary infection, these sites are often involved in cryptococcosis in HIV-negative, immunocompromised hosts.^3^ For this reason, we performed a pulmonary and CNS diagnostic work-up. High-resolution CT scan of the thorax showed multiple nodular opacities suggestive of pulmonary cryptococcosis (figure 1). Due to the absence of pulmonary symptoms, a respiratory specimen for microbiological investigation could not be obtained. Lumbar puncture revealed an elevated opening pressure (25 cm H_2_O, normal reference range=18–20). PCR on the cerebrospinal fluid (CSF) for cryptococcal antigen confirmed the presence of cryptococci in the CNS. Based on the presence of fungemia, infiltrative lung disease and CNS localisation, a diagnosis of disseminated cryptococcosis was established.

Because congenital or acquired deficiency of cell-mediated immunity usually underlies disseminated cryptococcosis, we performed flow cytometry to quantify and subclassify the patient’s lymphocytes. This revealed severe lymphopenia, with the complete absence of B cells and CD4^+^-T cells in the circulation. Additionally, the patient’s CD8^+^-T cell count was markedly diminished (50 cells/mm^3^, normal reference range=150–1000).

## Treatment

Idelalisib and prednisone were discontinued and antifungal therapy with liposomal amphotericin B (3 mg/kg intravenously, once daily) and flucytosine (25 mg/kg intravenously, four times daily) was initiated. Additionally, the patient received IVIGs to prevent relapse of haemolysis.

## Outcome and follow-up

**Figure 2:**
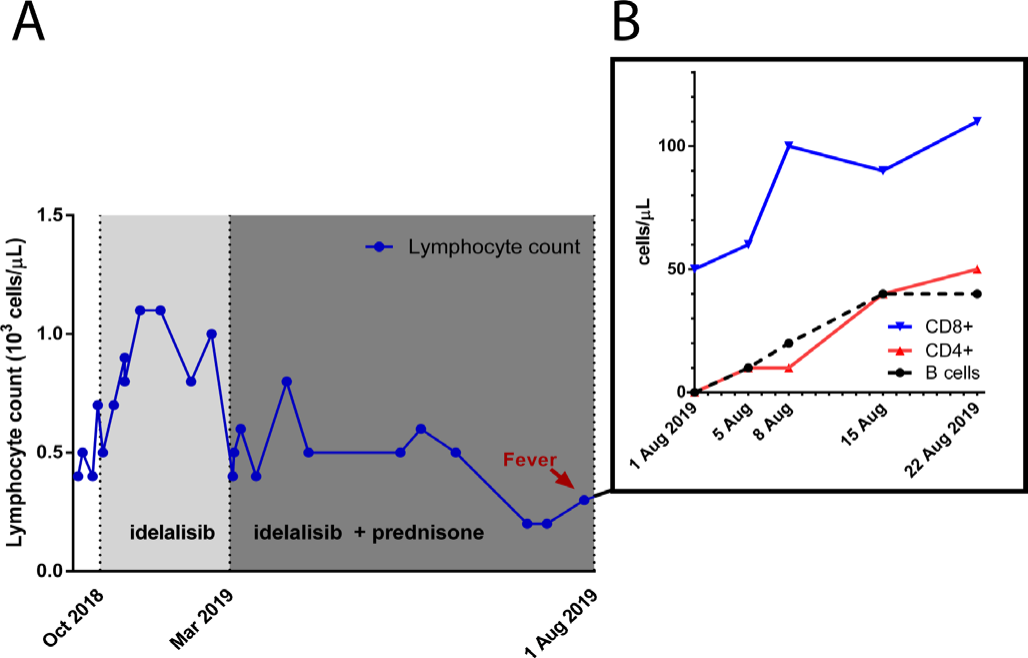
Counts of peripheral circulating lymphocytes. (A) Representation of the patient’s peripheral blood lymphocyte counts over time as measured by automated complete blood counting and leucocyte differentiation. (B) Representation of peripheral blood lymphocyte subset counts after withholding idelalisib and prednisone, as determined by flow cytometry (B cells, CD19^+^; T cells, CD3^+^; CD4^+^; CD8^+^ subsets).

Cessation of immunosuppressive therapy and initiation of antifungals were followed by the rapid but partial reconstitution of the lymphocyte subpopulations (see figure 2). The patient’s fever subsided, CRP levels diminished and on a repeat lumbar puncture, the CSF opening pressure had normalised and cultures of blood and CSF confirmed the absence of cryptococci. Following clinical improvement, the patient was discharged to his home with consolidative fluconazole therapy (400 mg orally one time per day) and IVIGs to prevent relapse of haemolysis. In adherence to clinical guidelines, we will continue consolidative therapy for the duration of 12 months.^4^

## Discussion

*C. neoformans* is an encapsulated environmental yeast that can cause life-threatening invasive infections in hosts with profoundly compromised cell-mediated immunity. The most common setting of invasive cryptococcosis is CD4^+^-T lymphopenia due to advanced HIV infection.^5^ Malignancy is also recognised as a predisposing risk factor, typically after treatment with lymphocyte-depleting chemotherapy.^2 6^ Interestingly, multiple cases of invasive cryptococcal disease in the absence of lymphopenia have recently been described in patients with chronic lymphocytic leukaemia (CLL) receiving treatment with ibrutinib, an oral tyrosine kinase inhibitor targeting Btk.^7^ It is hypothesised that this susceptibility is mediated through off-target inhibition of kinases in T lymphocytes or myeloid cells.^8^ However, to the best of our knowledge, invasive cryptococcal infection during idelalisib therapy has not previously been described.

Idelalisib, an inhibitor of the PI3K catalytic subunit δ isoform (PI3Kδ), targets intracellular signalling downstream of the B cell receptor and was approved in combination with rituximab for the treatment of CLL and as monotherapy for small lymphocytic lymphoma and FL.^9 10^ Paradoxically, adverse effects of idelalisib include both immunocompromise leading to opportunistic infections, most notably *Pneumocystis jirovecii* pneumonia and cytomegalovirus reactivation,^11^ but also autoimmune phenomena, including enterocolitis.^9^ Both immunosuppressive and autoimmune side effects of idelalisib are thought to be mediated through off-target inhibition of T lymphocyte biology, where PI3Kδ plays an important role in cellular activation and cytokine production.^12^ In an in vitro setting, it was demonstrated that regulatory T cells are especially sensitive to PI3Kδ inhibition, possibly explaining the autoimmunity associated with idelalisib treatment through the loss of self-tolerance.^13^ Additionally, in a mouse CLL model, inhibition of PI3Kδ reduced effector T cell function by inhibiting proliferation, activation, degranulation and production of cytokines, such as granzyme B and interferon gamma.^14^ This disruption of effector T cell biology could explain the increased susceptibility of patients on idelalisib to opportunistic infections generally associated with impaired T cell function. Consequently, the European Society of Clinical Microbiology and Infectious Diseases recommends prophylaxis with trimethoprim-sulfamethoxazole during idelalisib therapy.^15^

This patient’s severe lymphopenia with complete B cell depletion and significant T cell suppression likely underlied his susceptibility to invasive cryptococcal infection. Although idelalisib interferes with T cell biology, depletion of circulating T lymphocytes is unusual. Indeed, in the original phase II study evaluating idelalisib monotherapy for FL, no significant changes in T cell subpopulations were reported.^10^ Concomitant long-term corticosteroid use likely contributed to the immunocompromised state. This is supported by the observation that lymphocyte counts gradually, but persistently dropped after initiating corticosteroids alongside idelalisib (figure 2). Corticosteroid administration causes lymphopenia, mainly due to redistribution of lymphocytes to secondary lymphoid sites.^16^ Corticosteroids predominantly deplete T cells, whereas B cells are affected to a lesser extent.^17^ Moreover, long-term corticosteroid use has previously been associated with invasive cryptococcal infection in HIV-negative individuals.^18^ Due to the rapid partial reconstitution of lymphocyte numbers after withholding idelalisib and prednisone, we consider alternative causes of lymphopenia, such as bone marrow failure or paraneoplastic destruction, to be unlikely (figure 2).

In conclusion, we describe a case of disseminated cryptococcosis affecting a patient with severe lymphopenia in the setting of combination therapy with idelalisib and prednisone for follicular lymphoma. Cessation of immunosuppressive therapy and administration of systemic antifungal agents led to the rapid partial repletion of lymphocyte counts and a favourable clinical outcome. Our clinical case highlights that idelalisib in combination with corticosteroids, although developed for the treatment of clonal B cell disease, can significantly suppress T cell-mediated immunity and additionally we provide an estimate of the kinetics of lymphocyte reconstitution after withholding idelalisib and corticosteroids. Physicians should be aware of the risk of various opportunistic infections in patients treated with idelalisib and other novel targeted therapies in combination with corticosteroids for FL or CLL.

**Learning points**

- Small molecular inhibitors for the treatment of indolent B cell neoplasia, such as idelalisib, can cause significant immunocompromise.
- Physicians should be aware of the risk of various opportunistic infections in patients treated with such drugs.
- Patients treated with idelalisib and corticosteroids are at risk for lymphopenia and infection with *Cryptococcus neoformans*
- B cell and T cell compartments reach 50% of normal levels within 1 month after abrupt withdrawal from idelalisib and corticosteroids.
